# Evaluation of nephroprotection of silymarin on contrast-induced nephropathy in liver cirrhosis patients: A population-based cohort study

**DOI:** 10.1097/MD.0000000000012243

**Published:** 2018-09-14

**Authors:** Yu-Jui Kuo, Hui-Ping Chang, Yu-Jun Chang, Hsing-Hsien Wu, Chang-Hua Chen

**Affiliations:** a Department of Traditional Chinese Medicine, Tainan Municipal Hospital (Managed by Show Chwan Medical Care Corporation); b Department of Applied Cosmetology, National Tainan Junior College of Nursing Tainan; c Epidemiology and Biostatistics Center, Changhua Christian Hospital, Changhua; d Department of Thoracic Surgery, Tainan Municipal Hospital (Managed by Show Chwan Medical Care Corporation); e Center of Infection Prevention and Control; f Department of Internal Medicine, Changhua Christian Hospital, Changhua; g Ph.D. Program in Translational Medicine, National Chung Hsing University, Taichung; h Rong Hsing Research Center for Translational Medicine, National Chung Hsing University, Taichung, Taiwan.

**Keywords:** contrast-induced nephropathy, population-based cohort study, silymarin

## Abstract

Recent findings from an animal experiment suggest a modest association between silymarin and decreased risk of contrast-induced nephropathy. However, the relationship between silymarin and contrast-induced nephropathy in patients with liver cirrhosis remains unclear.

From 1997 to 2007, we identified 3019 patients with liver cirrhosis who were administered silymarin and matched them with 3019 patients with liver cirrhosis who were not administered silymarin. Each patient was followed up for a minimum of 4 years. After adjusting for age, gender, hepatitis B, hepatitis C, alcoholic hepatitis, and Charlson comorbidity index, we considered death occurrence and used the Fine and Gray regression models to calculate subdistribution hazard ratios (sHRs) for contrast-induced nephropathy. Sensitivity analyses were also performed using the same model on the subgroups classified by comorbidity.

Using the Fine and Gray regression models and with death as the competing risk, we observed that sHR for contrast-induced nephropathy was 0.94-fold higher in the silymarin cohort than in the nonsilymarin cohort (95% confidence interval = 0.61–1.47, *P* = .791). On the basis of sensitivity analyses results classified by comorbidity, a nonsignificant decrease in risk of contrast-induced nephropathy was found.

Silymarin shows no nephron-protective positive effects on contrast-induced nephropathy. Silymarin did not play a nephron-protective role according to Longitudinal Health Insurance Database of Taiwan. Clinical trials are necessary to further assess the nephron-protective effects of silymarin of contrast-induced nephropathy.

## Abstract 2

Supplemental Digital Content is available in the text

## 1 Introduction

Contrast medium (CM) is one of the most common pharmacological agents injected in hospitalized patients.^[1]^ Considering the increasing number of patients undergoing computed tomography (CT),^[2,3]^ many more patients experienced CM-related adverse events (AEs). CM-adverse reactions (ARs) and those originating from mild symptoms can potentially be life-threatening. Although low-osmolarity nonionic CMs have been introduced since the mid-1970s to reduce CM-ARs, CM-ARs have still been reported.^[3–5]^ CM-induced nephrotoxicity (CMIN) is one of the major causes of acute kidney injury (AKI) among hospitalized patients. CM-ARs cannot always be predicted, but various studies indicated that CMIN pathophysiology is closely related to renal hemodynamic changes and medullary ischemic injury, reactive oxygen species (ROS)-induced oxidative stress damage, indirect damage to the tubules, and tubular obstruction.^[6,7]^ Among the possible pathogenesis mechanisms of CMIN, ROS-induced oxidative stress damage is important.^[7,8]^ It is currently an important target for drug intervention to prevent CMIN. To decrease and prevent CM-ARs, several guidelines have been developed to prevent AEs, but these guidelines are only partially successful.^[9,10]^ Limited evidence prove the effectiveness of premedication before CM administration.^[11]^

Silymarin is a hepatoprotective drug.^[12–14]^ Two major mechanisms have been proposed to account for the organ-protective effects of this compound. The first mechanism is its dose-dependent antioxidant effect.^[15]^ The second mechanism involves its anti-inflammatory and antiapoptotic properties.^[15]^ Silymarin may act as a nephron-protective agent against CMIN.^[12]^ To date, the protective effects of silymarin on CMIN have been primarily investigated in animals, and nephroprotection was observed. However, large-scale clinical observations are needed to prove the nephroprotection effects of silymarin.

Among possible pathogenesis mechanisms of CMIN, ROS-induced oxidative stress damage is one of the most important.^[8]^ N-acetylcysteine has been recognized as a CMIN prevention drug because of its strong antioxidant effects that can prevent CMIN.^[7,11]^ However, N-acetyl cysteine may slow down the blood clotting, and patients receiving CT examination require a large-sized needle for CM injection. Silymarin possesses both antioxidative and anti-inflammatory effects and is commonly used to manage hepatitis. However, few evidence prove the nephroprotective effect on CMIN. The current study aimed to determine and evaluate the nephroprotective effect of silymarin on CMIN cohorts from the longitudinal National Health Insurance Research Database.

## 2 Methods and materials

### 2.1 Data sources and study subjects

Silymarin and nonsilymarin cohorts were obtained from the Longitudinal Health Insurance Databases (LHIDs), including LHID2000, LHID2005, and LHID2010. LHID2000, LHID2005, and LHID2010 included all the original claim data randomized from the beneficiary registry in 2000, 2005, and 2010, respectively, and the registration file of 1 million individuals (N = 23.72 million) for the Taiwan National Health Insurance (NHI) program. According to the National Institutes of Health in Taiwan, no significant difference was found in the gender distribution of enrolled students and the list of enrolled students under the National Health Plan for enrollment opportunities for undergraduates throughout the country. The LHID enables researchers to access all medical services provided to individuals registered in the database from the beginning of the 1995 NHI. Such data can be used to explore the link between silymarin and contrast-induced nephropathy. The study was expelled from the Tainan Municipal Hospital Authority Review Board because it used LHID2000, LHID2005, and LHID2010, which included secondary data released to the public for research purposes. This study was approved by the Tainan Municipal Hospital.

Patients with liver cirrhosis (international classification of diseases, 9th revision diagnostic codes 571.5 and 571.6) who were identified between 1997 and 2007 were selected from the database. For inclusion, at least one of the following criteria should be met: diagnosis of cirrhosis of one or more hospitalized patients; and diagnosis of liver cirrhosis at 3 or more outpatient visits within 6 months. Index day for the patients with liver cirrhosis was assigned as 1 year after the newly liver cirrhosis diagnosis. Prescribed use of silymarin medications in the follow-up period was also considered. Prescription records contained dates of order, dosage, route of every prescription, and number of days. Two cohorts were categorized from the patients with liver cirrhosis. The first cohort included patients who regularly use silymarin medication (silymarin cohort). The other cohort included patients who did not use any silymarin medication (nonsilymarin cohort) during the follow-up period. The nonsilymarin cohort was matched (1:1) with the silymarin cohort according to age, gender, Charlson comorbidity, and index day. Patients with diagnosis of contrast-induced nephropathy prior to the index day were excluded from the study. Comorbidities were classified as those existing prior to the index day and included Charlson comorbidity, hepatitis B, and hepatitis C. The study also categorized liver cirrhosis into alcoholic and nonalcoholic types. The end of the follow-up period for the analyses was marked on the day of contrast-induced nephropathy diagnosis and terminated on 2012 or upon death. Follow-up data were available for a minimum of 4 years for all selected subjects.

### 2.2 Contrast-induced nephropathy

In this study, the definition of contrast-induced nephropathy is combine receiving CT examination (computerized tomography code) and exposure to contrast (contrast code) and within 1 week duration between the date of new nephropathy diagnosis (nephropathy code) and contrast exposure. The source code is listed at Appendix.

### 2.3 Statistical analysis

The study used the *t* test for continuous variables and chi-squared test for categorical variables to analyze the differences between silymarin and nonsilymarin cohorts. The baseline characteristics from the database included age, gender, Charlson comorbidity, hepatitis B, hepatitis C, and alcoholic liver cirrhosis. The number of contrast-induced nephropathy cases in the 2 cohorts during follow up was counted. The subdistribution hazard ratio (sHR) was calculated using the Fine and Gray competing risk regression models, whereas a regression hazard model was used to compare the silymarin and nonsilymarin cohorts to assess the risk of contrast-induced nephropathy. Kaplan–Meier method was used to determine the cumulative incidence of CMIN in both cohorts, and differences between cohorts were tested using the Gray test. To examine whether the main findings had different assumptions, sensitivity analyses were performed. Sensitivity analyses were also performed using the Fine and Gray regression hazard models on subgroups classified by comorbidity. All data management and sHR calculations were conducted using Statistical Analysis System (SAS) software for Windows (version 9.4; SAS Institute, Cary, NC).

## 3 Results

**Figure 1:**
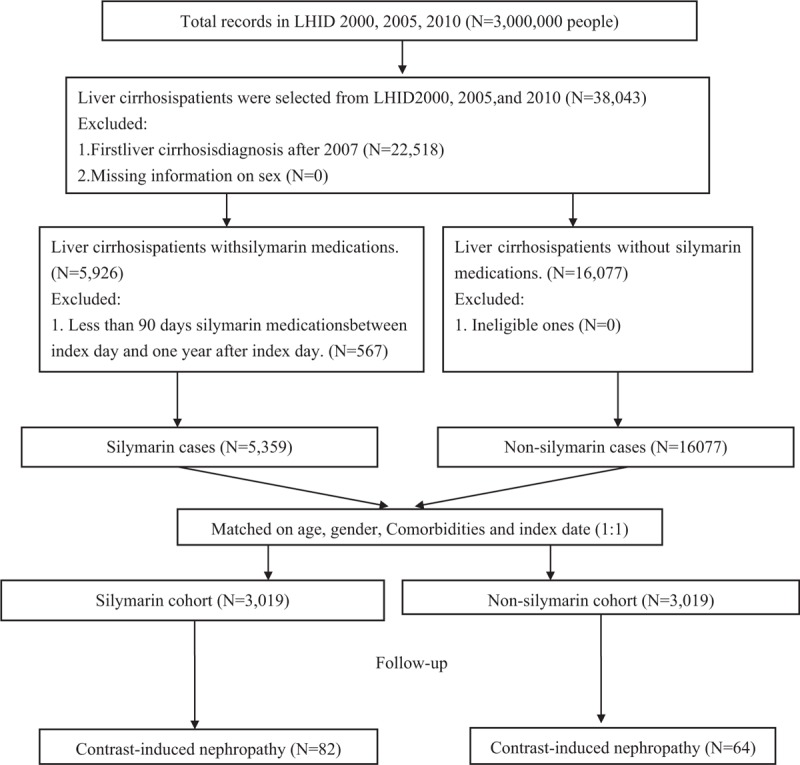
Flow chart of study subjects selection in this study from longitudinal National Health Insurance Research Database. LHID = Longitudinal Health Insurance Database.

**Figure 2:**
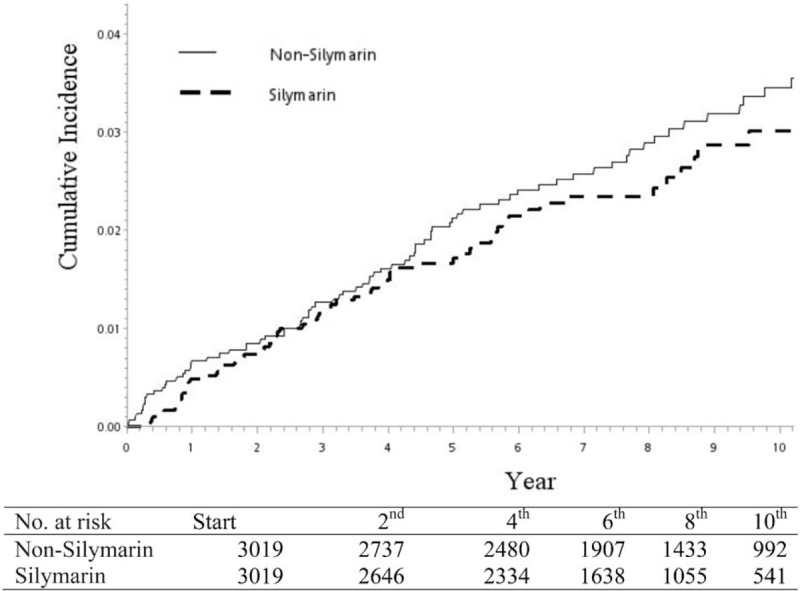
Cumulative incidences of contrast-induced nephropathy for silymarin cohort and matched nonsilymarin cohort.

The silymarin cohort included 3019 patients identified from January 1, 1997 to December 31, 2007. Meanwhile, 3019 subjects who were not receiving silymarin medications at baseline were randomly assigned to the nonsilymarin cohort with age, Charlson comorbidity index (CCI), and index days after excluding unqualified subjects (Fig. 1). After matching, the age, gender, and CCI comorbidity distributions were found to be similar between the silymarin and nonsilymarin cohorts (Table 1). Most subjects were 40 to 59 years old or 60 to 79 years old, and these age groups agreed with the characteristics of contrast-induced nephropathy. Kaplan–Meier curves showed that the cumulative incidence of contrast-induced nephropathy in the silymarin cohort was nonsignificantly lower than in the nonsilymarin cohort (Fig. 2). The risk of contrast-induced nephropathy in the silymarin patients was 0.94 (95% confidence interval = 0.61–1.47, *P* = .791) after adjusting for age, gender, hepatitis B, hepatitis C, alcoholic liver cirrhosis, and CCI in the stratified Fine and Gray models (Table 2). Kaplan–Meier curves showed that the cumulative incidence of contrast-induced nephropathy in the silymarin cohort was nonsignificantly lower than in the nonsilymarin cohort (Fig. 2). From Table 3, the study also found the nonsignificant results between the 2 cohorts among all selected comorbidities.

**Table 1:**
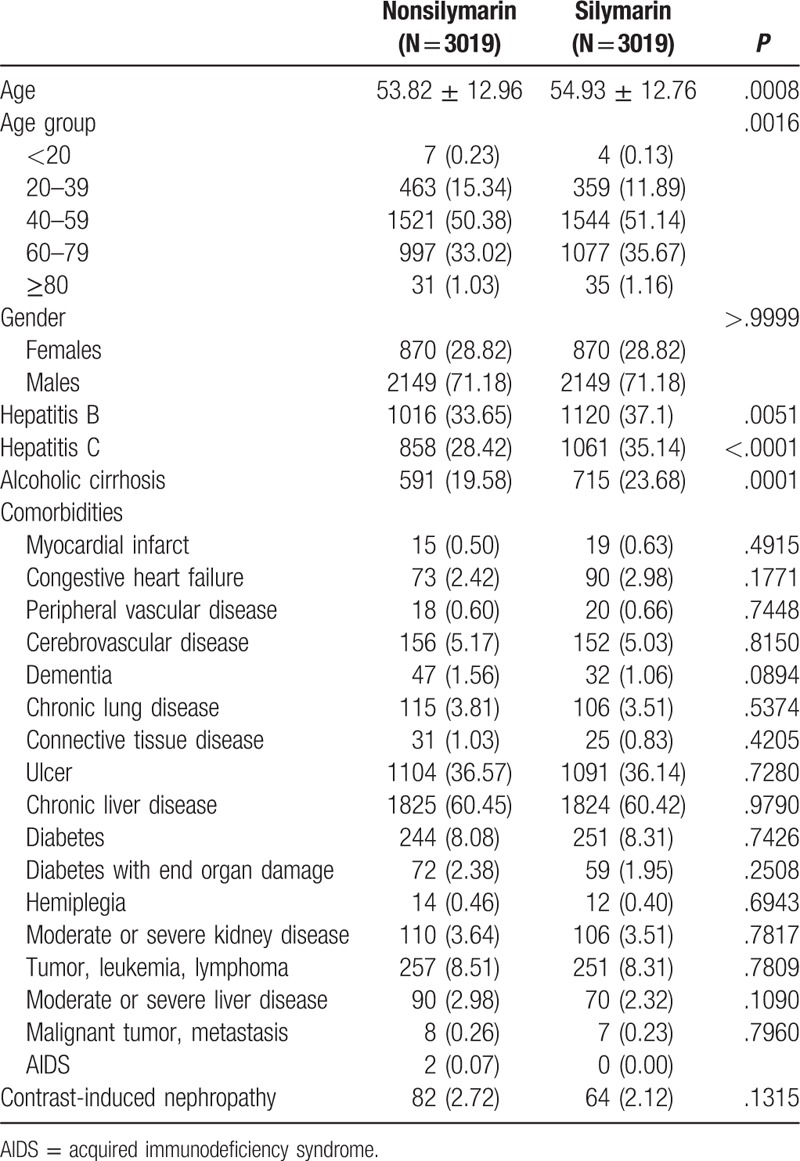
Characteristics of study subjects selection in this study from longitudinal National Health Insurance Research Database.

**Table 2:**
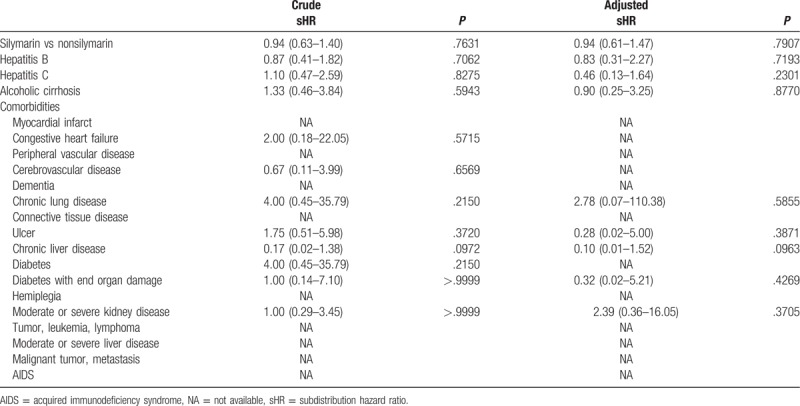
Prediction for occurrence of contrast-induced nephropathy in this study from longitudinal National Health Insurance Research Database.

**Table 3:**
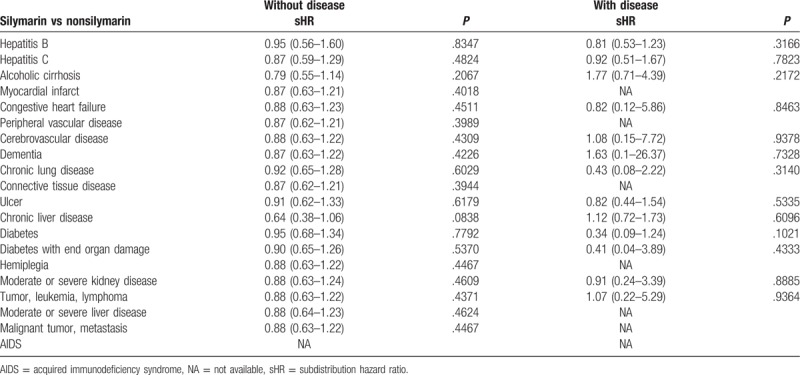
Comparison between the 2 cohorts among all selected comorbidities in this study from longitudinal National Health Insurance Research Database.

## 4 Discussion

This work is the first nationwide, population-based follow-up study that determined whether silymarin exerts significant nephron-protective effects on patients with CMIN. Hospital-acquired AKIs, including CMIN, are important causes of mortality and morbidity. Several CMIN treatment options have been proposed.^[6]^ However, CMIN remains a major problem for health care. Silymarin shows no nephroprotective role according to this population-based, nested case–control study.

The incidence of CMIN ranges between 2% and 7%.^[3,16,17]^ CM-AR rates range between 0.7% and 0.82%.^[1,3,18,19]^ However, studies rarely reported nephrotoxic CM-ARs, and the possible reasons for result include aggressive premedication and hydration before CT examinations among high-risk patients. The total incidence of CMIN is low. In the current study, such low value may indicate the negative nephroprotection effect of silymarin on CMIN.

Silymarin is a useful hepatoprotective medication because of its antioxidant and anti-inflammatory properties.^[14,20,21]^

Theoretically, silymarin may positively affect patients with CMIN. Silymarin decreased renal damage and restored ROS activities in an animal model.^[22]^ Dashti-Khavidaki et al reported the nephroprotective effects of silymarin against some nephrotoxins.^[14]^ Khan et al reported that silymarin treatment can increase kidney weight from renal damage status.^[23]^ Kaur et al reported the potent nephroprotective effect of silymarin in an animal mode.^[24]^

However, silymarin can exacerbate renal damage in an animal model according to the study of Homse et al.^[25]^ The study of Homse et al revealed that silymarin can result in persistent oxidative stress and inflammatory processes, tubular necrosis, and apoptosis.^[25]^

In our study, silymarin did not play a nephroprotective role. This finding might have been affected by the following: inadequate patient numbers, inadequate dosage and duration, and inaccurate prescription timing. Further studies are needed in the future to evaluate the nephroprotective effects of silymarin against CMIN.

### 4.1 Limitations

The current study used the Taiwan NHI database, which includes data from a longitudinal cohort and is a large and population-based database. The nationwide LHID 2000 is an excellent resource for evaluating patients with CMIN. Our study is relevant because it evaluated the nephroprotective effects of silymarin against CMIN.

Some limitations were considered. First, several CMIN patients were not reported in LHID, and we assumed that the dataset from the NHI program are relatively accurate. Second, laboratory information about some potential bias, including coding bias, was lacking. Third, no laboratory data are available in the NHI Research Database. Therefore, we cannot determine the severity of CMIN in our current patients.

In conclusion, silymarin did not exert nephroprotective positive effects on CMIN. Although CMIN remains a burden among hospitalized patients, silymarin cannot be recommended as a nephron-protective drug. After reviewing the major studies focusing on the role of silymarin in nephroprotection, silymarin administration to animals can reduce or prevent CMIN. However, silymarin did not exhibit any nephroprotective role according to the LHID of Taiwan. Further clinical trials are necessary to assess the nephron-protective effects of silymarin on CMIN.

## Author contributions

**Conceptualization:** Yu-Jui Kuo, Hui-Ping Chang, Yu-Jun Chang, Hsing-Hsien Wu, Chang-Hua Chen.

**Data curation:** Hui-Ping Chang, Chang-Hua Chen.

**Formal analysis:** Yu-Jun Chang.

**Funding acquisition:** Chang-Hua Chen.

**Methodology:** Yu-Jun Chang.

**Project administration:** Chang-Hua Chen.

**Supervision:** Hsing-Hsien Wu, Chang-Hua Chen.

**Validation:** Yu-Jun Chang, Hsing-Hsien Wu, Chang-Hua Chen.

**Writing – original draft:** Yu-Jui Kuo, Chang-Hua Chen.

**Writing – review & editing:** Yu-Jui Kuo, Hui-Ping Chang, Yu-Jun Chang, Hsing-Hsien Wu, Chang-Hua Chen.

## Supplementary Material

Supplemental Digital Content
