# Supplementary material for: Evaluation of nephroprotection of silymarin on contrast-induced nephropathy in liver cirrhosis patients: A population-based cohort study
Source: Medicine (Baltimore). 2018 Sep 14;97(37):e12243. doi: 10.1097/MD.0000000000012243 (PMC6155955; doi:10.1097/MD.0000000000012243)
Supplement: Supplemental Digital Content [file medi-97-e12243-s001.docx]

**Appendix 1. The definition of contrast-induced nephropathy**

**The definition of contrast-induced nephropathy is combine receiving computerized tomography examination (computerized tomography code) and exposure to contrast (contrast code) and within one week duration between the date of new nephropathy diagnosis ( nephropathy code ) and contrast exposure. The source code is listed as follows:**

**Computerized tomography Code**

Other computerized axial tomography (ICD= 88.38)

**Contrast Code**

1. Other and unspecified drugs (ICD9 = E947), medicinal substances (ICD9 = E947.8), and other drugs and medicinal substances (Contrast media used for diagnostic x-ray procedures Diagnostic agents and kits)
2. Excluded poisoning by other and unspecified drugs (ICD9 = 977), medicinal substances(ICD9 = 977.8), and other specified drugs and medicinal substances (Contrast media used for diagnostic x-ray procedures Diagnostic agents and kits)

**Nephropathy Code**

Nephritis, Nephrotic Syndrome, And Nephrosis ( ICD=580-589).
